# Executive cognitive ability and business model innovation in start-ups: The role of entrepreneurial bricolage and environmental dynamism

**DOI:** 10.3389/fpsyg.2022.978543

**Published:** 2022-08-25

**Authors:** De'en Hou, Aihua Xiong, Chen Lin

**Affiliations:** ^1^School of Business Administration, Shandong University of Finance and Economics, Jinan, China; ^2^School of Economics and Management, Nanjing University of Science and Technology, Nanjing, China

**Keywords:** executive cognitive ability, business model innovation, environmental dynamism, entrepreneurial bricolage, entrepreneurial success

## Abstract

Extant literature suggested that executive cognitive ability is a critical perspective to answering why and how enterprises perform business model innovation. However, the effect of executive cognitive ability on business model innovation is still insufficiently explored. Drawing on entrepreneurial bricolage theory, we developed a moderated mediation model which takes entrepreneurial bricolage as the mediating mechanism and environmental dynamics as the moderating mechanism to explain how executive cognitive ability influences business model innovation. We collected the data of 316 executives of Chinese start-ups through questionnaires for the model test. Results showed that new venture executives' cognitive ability significantly positively affects business model innovation by mediating with entrepreneurial bricolage. Environmental dynamism positively moderates the effect of executives' cognitive ability on business model innovation. Moreover, environmental dynamism positively moderates the mediating role of entrepreneurial bricolage in executive cognitive ability and business model innovation. This study broadens the research scope of entrepreneurial bricolage theory from the perspective of cognitive ability and provides ideas for new ventures' business model innovation.

## Introduction

The rapid development of the digital economy has given rise to a boom in global entrepreneurship, which has promoted massive entrepreneurial opportunities (Herve et al., 2020). According to the Global Entrepreneurship Monitor's 2019 global report, there are more than 10 million registered enterprises every year, showing a growth trend (Bosma and Kelley, 2019). In the past 5 years, China has become one of the most active countries in innovation and entrepreneurship. The development of mass entrepreneurship and innovation activities has further stimulated the vitality of the national economy (Wu and Zhang, 2021). However, while innovation and entrepreneurship activities are highly concerned, the low survival rate of enterprises caused by the change in the entrepreneurial environment and innovation dilemma cannot be ignored. According to statistics, more than 2/3 of new ventures in China have lasted for less than 5 years (Wu and Zhang, 2021). The dilemma of new ventures' survival and development is usually complex. Clausen (2020) found that financing difficulties, lack of resources and fierce competitive markets are important factors, and insufficient business model innovation is the core reason. Therefore, business model innovation has become the key factor for new ventures to survive in the market, but how new ventures carry out business model innovation has not been effectively explored (Anwar, 2018). Accordingly, analyzing the influencing mechanism of new ventures' business model innovation is necessary. In the complex and changeable environment, how new ventures solve the problems of survival and development through business model innovation? How executives find effective ways of business model innovation? These are the key issues that new ventures must consider and the key issues to discuss in this study.

As start-up leaders, executives can directly impact innovation decisions (Nadkarni and Barr, 2008). New venture executives make innovation decisions that are not arbitrary but usually based on their cognitive ability to explore the external environment and innovation opportunities, then form strategic decisions and allocate enterprise resources (Sarasvathy, 2004). In innovation decision-making, new venture executives conduct an environmental analysis and identify innovation opportunities based on unique cognitive ability, which is an important aspect affecting the business model innovation (Zhou et al., 2021). Especially in a dynamic environment, the cognitive ability of executives is very beneficial to business model innovation. Executives with favorable cognitive ability can fully identify risks in dynamic environments and develop valuable business model innovation opportunities. Ultimately, more stakeholders will be recognized and enterprises will gain growth momentum (Shepherd et al., 2015). Therefore, this study seeks to clarify the effect of executives' cognitive ability on business model innovation.

When considering the impact of executive cognitive ability on business model innovation, the research discussed innovation opportunities, entrepreneurial environment, and entrepreneurial team building. Osiyevskyy and Jim (2015) found that identifying innovation opportunities is an essential prerequisite for business model innovation, and it is necessary to analyze and grasp heterogeneous opportunities. Baron and Henry (2010) pointed out that executive cognitive ability can promote the entrepreneurial team's comprehension of the entrepreneurial environment, help identify critical resource gaps restricting enterprise development, and build knowledge and resource acquisition mechanisms. Amit and Zott (2015) believe that business model innovation depends on entrepreneurs integrating and analyzing innovation opportunities and business elements systematically, reflecting the importance of entrepreneurial cognitive ability.

The existing research on the impact of cognitive ability on business model innovation is still deficient, which is not conducive to revealing the law behind the subjectivity of the innovation process and behavior representation of new ventures (Venkataraman et al., 2012; Shepherd et al., 2015). The mechanism is also an important research issue to be explored in entrepreneurship research. In addition, some scholars believe it is necessary to explore the impact of executive cognitive ability on enterprise innovation decision-making in the Chinese business environment and cultural background (Hu and Jiao, 2019). Therefore, this study attempts to explore the impact of executive cognitive ability on business model innovation in the context of China.

**Figure 1:**
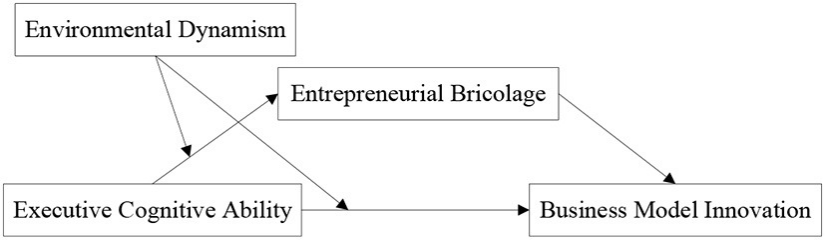
Hypothesized model.

The mediating and moderating mechanisms discussed in this study are inspired by entrepreneurial bricolage theory. The entrepreneurial bricolage theory can explain how managers break the traditional resource and environment analysis paradigm and reshape enterprises' resource endowment through bricolage. It can also explain how new ventures cope with the challenges brought by environmental uncertainty and resource dilemmas. Scholars have pointed out that new venture managers obtain the resources needed for business model innovation through entrepreneurial bricolage (Baker and Nelson, 2005), which can be regarded as business model innovation in a resource-constrained environment. This course can also explain the relationship between executive cognitive ability, resource acquisition, and business model innovation under resource constraints. Therefore, we propose that entrepreneurial bricolage plays an intermediary role in executive cognitive ability and business model innovation. Moreover, although recent studies have shown that environmental dynamism affects entrepreneurial bricolage and innovation (Yan et al., 2020), we still do not know how environmental dynamism affects the relationship between executive cognitive ability and business model innovation. Environmental dynamism is a vital contingency factor affecting the effectiveness of enterprise innovation. Its unforeseen characteristics affect the critical aspects of enterprise business philosophy and bring varying degrees of impact on enterprise resource acquisition, integration, and innovation. (Yan et al., 2020). Therefore, our research believes that environmental dynamism is vital in determining the relationship between executives' cognitive ability, entrepreneurial bricolage, and business model innovation. The complete research model is shown in Figure 1.

This study aims to explore the mechanisms between new ventures' executive cognitive ability and business model innovation in the Chinese context. Firstly, the relationship between executive cognitive ability and business model innovation is tested. Then entrepreneurial bricolage is introduced as the mediating variable, and environmental dynamism is introduced as the moderating variable to verify the moderated mediating effect of executive cognitive ability on business model innovation. This study will broaden the research scope of entrepreneurial bricolage theory and enable new venture executives to integrate resources through entrepreneurial bricolage and improve the efficiency of business model innovation.

## Literature review and hypotheses

### The direct influence of executive cognitive ability on business model innovation

The entrepreneurial environment is always complex and changeable. Business model innovation (BMI) is essential for new ventures to adapt to the environment. The business model innovation activities of new ventures reflect the cognitive process of executives' processing and interpretation of internal and external environmental information (Helfat and Peteraf, 2015). In business model innovation, executives often need to integrate and analyze opportunities and business elements systematically, reflecting the importance of executive cognitive ability (Amit and Zott, 2015).

New ventures' executive cognitive ability (ECA) is an important aspect affecting business model innovation. In an uncertain environment, executives realize business model innovation by promoting value creation logic (Shook, 2003). However, without mature experience, executives' cognitive ability plays an vital role in business model innovation (Bonesso et al., 2018). In the changing environment, the executives' cognitive ability helps new ventures accurately grasp customer needs and assay market development trends (Narayan et al., 2021). Finally, it helps enterprises clarify their market position and determine business model innovation goals (Eggers and Kaplan, 2009). Narayan et al. (2021) found that cognitive ability positively impacts innovation from the perspective of social behavior. In addition, Salas and Fiore (2004) found that cognitive ability plays an essential role in team collaboration. Teamwork with high cognitive ability is conducive to improving innovation performance (Orlando et al., 2002). Helfat and Peteraf (2015) pointed out that CEO's cognitive ability is very beneficial to business model innovation. Therefore, the more intense the new venture executives' cognitive ability, the greater possibility of new ventures carrying out business model innovation. Based on this, we propose the following hypothesis:

H1: ECA is positively associated with BMI in start-ups.

### Mediating role of entrepreneurial bricolage

Bricolage has become an innovation strategy adopted by SMEs in a highly competitive and volatile environment, which helps enterprises to achieve innovation in value creation, value proposition, and value acquisition (Zott et al., 2011). New ventures often face resource constraints due to their newborn weakness, which hinders their development of business model innovation (Yang, 2018). Entrepreneurial bricolage (EB) is a path for new ventures to integrate resources (Cui and Pan, 2015), which provides adequate support for new ventures to carry out business model innovation under resource constraints (Banerjee and Campbell, 2009). Entrepreneurial bricolage can help enterprises enrich the space of resource utilization and establish new resource allocation logic. In particular, creative resource reorganization can solve the problem of resource scarcity in new enterprises (Baker and Nelson, 2005). When new ventures' executives are aware of the resource constraints, they will make a top-level design to break the resource constraints and obtain the scarce resources needed for business model innovation through entrepreneurial bricolage (Massa et al., 2017). At the same time, when executives realize that the current resource mix of enterprises does not have environmental adaptability and cannot promote business model innovation, they will also re-optimize resource structure through entrepreneurial bricolage (Baden-Fuller and Haefliger, 2013).

Entrepreneurial bricolage plays a bridge role between executive cognitive ability and business model innovation (Baker and Nelson, 2005). On the one hand, enterprises can integrate existing and new resources through bricolage, form new value propositions, and help enterprises expand market boundaries (Yan et al., 2020). At the same time, the bricolage process helps enterprises break the traditional industry rules, form new regulations and achieve remodeling. On the other hand, through bricolage, enterprises can reshape the existing resource endowment and utilization model, break through the resource bottleneck restricting innovation activities, and promote the business model innovation of new ventures (Yan et al., 2020). One of the most advanced studies also shows that cognitive ability can enhance the ability of managers to integrate internal and external resources, help enterprises identify new business opportunities, and promote business model innovation (Amit and Zott, 2015). In addition, entrepreneurial bricolage helps executives to strengthen rational analysis of existing resource attributes and defects, deepen the understanding of resource integration methods, and further stimulate new ventures' business model innovation (Senyard et al., 2014). Based on this, the following hypotheses are proposed:

H2: The positive association of ECA on BMI is mediated by EB.

### Moderating role of environmental dynamism

Existing research mainly discusses the influence of entrepreneurial orientation, cognitive flexibility, and relational embeddedness on entrepreneurial bricolage. Studies have shown that entrepreneurial orientation and bricolage can fit each other (Baker et al., 2003). Cognitive flexibility helps enterprises to carry out bricolage to solve problems (Canas et al., 2003). Entrepreneurial learning helps entrepreneurs gain piecemeal experience. In addition, relational embeddedness helps new ventures maximize the resources in interpersonal networks and improve bricolage effectiveness (Dirk et al., 2016). Existing research attempts to discuss the factors that stimulate entrepreneurial bricolage behavior and efficacy from different perspectives, but the current research has not fully paid attention to the impact of environmental dynamics on the entrepreneurial bricolage. This study attempts to reveal the impact of environmental dynamics on entrepreneurial bricolage.

The external environment of enterprises includes many factors such as government policy, market demand, technological change, and operational risk, which are essential conditions affecting the survival and growth of new ventures (Rosenbusch et al., 2013). When environmental dynamics (ED) increase, the connection between the various elements of the external environment becomes blurred, which increases the difficulty of new ventures' business model innovation. In such a problematic situation, new ventures with solid executives' cognitive ability tend to have a more apparent entrepreneurial orientation and will quickly identify the technological and market risks caused by environmental changes (Bogner and Barr, 2000). Managers interact with the environment through innovation opportunity identification to find critical elements of business model innovation (Futterer et al., 2018). Wofford (1994) found that managers with high cognitive ability can predict unknown problems more accurately and solve complex problems can form more creative ideas. Yasemin and Mesko (2013) found that a management team with high cognitive ability can accurately make effective management decisions, and ultimately achieve management goals. Osiyevskyy and Jim (2015) found that entrepreneurs' perception of opportunities, threats, and risk experiences will affect the innovation intention of new ventures' business models.

In addition, environmental dynamism increases the difficulty of obtaining innovative resources, resulting in new ventures' inability to obtain sufficient resources, hindering the smooth development of business model innovation activities. Faced with resource constraints, new venture executives can follow the bricolage principle of good decision-making or improvisational decision-making to cope with changes in the external environment by patching resources (Senyard et al., 2009). Putting the most appropriate resources or resource mix into key activities helps generate new products and services, which primarily solves the resource shortage dilemma of new ventures. Thomke (1997) found that new ventures face high environmental dynamics and resource scarcity, and executive cognitive ability can help identify entrepreneurial opportunities in transforming environmental challenges. Executives solve the problem of resource shortage in business model innovation by guiding enterprises to carry out entrepreneurial bricolage and applying a suitable combination of resources to critical businesses. Based on this, the following hypotheses are proposed:

H3: ED moderates the positive relationship between ECA and BMI; the higher the ED, the more significant the positive influence of ECA on BMI.

H4: ED moderates the mediating association of EB between ECA and EB; the higher the ED, the more significant the mediating association of EB between ECA and BMI.

## Methods

### Sample and procedures

China's unique entrepreneurial and innovative practices provide a unique context for researchers. From the perspective of environmental dynamics, China has continuously invested in technology, management, and business models of emerging industries in recent years, creating a more dynamic environment through a bottom-up approach. From the perspective of entrepreneurial bricolage, culture and informal institutions such as “harmony” in the Chinese context provide opportunities for developing entrepreneurial bricolage theory. Therefore, this study chooses China as a unique context.

This study uses simple random sampling to ensure that each new venture has the same opportunity to be drawn (Rahman et al., 2022). The research data is obtained by questionnaire survey, mainly through the website “Wen Juanxing” and field paper questionnaire survey. The Chinese enterprises selected in the research must be new enterprises with a business license or formal operation for more than half a year and generally not more than 8 years (Zahra and Bogner, 2000). In order to avoid the problem of homologous variance caused by the questionnaire survey, we mainly do the following work. Firstly, before the questionnaire is issued, the respondents must fill in anonymously and be told the data is only for academic research. Secondly, to avoid the respondents' interference, we investigated the data of no more than three executives in each new venture. Finally, it limits the repeated access to internet protocols and avoids the problem of repeated filling. The survey ended after receiving 500 respondents. A total of 382 questionnaires were left after excluding the wrong-filled and open-filled questionnaires. We exclude 66 questionnaires with too short or too long answer times according to the statistics of “Wen Juanxing.” Finally, 316 valid questionnaires were obtained, and the recovery rate of effective questionnaires was 63.2%. This study conducts a deviation test on the collected effective samples from the years of establishment of new ventures, enterprise size, age of the enterprise (year), and educational background and age of enterprise executives. The results show that deviation problem is not apparent, which aligns with relevant regulations. The research enterprises were established between 2–6 years (87.6%). The respondents are between 21 and 40 years old (96.5%). More than 80% of respondents have a bachelor's degree (87.5%). The respondents came from enterprises with less than 50 employees (80.5%).

### Measures

In order to ensure the reliability and validity of the data, this study selects authoritative journal scales to design the questionnaire. All English scales adopt double-blind translation procedures to avoid semantic ambiguity to the greatest extent. The questionnaire design includes scale selection and double-blind translation, structured interviews, pre-research, and questionnaire revision. In the process of double-blind questionnaire translation, one doctor translates English into Chinese, and the other doctor translates Chinese into English in a back-to-back way. Finally, a professional professor and a language professor proofread the questionnaire. In the structured interview, eight new ventures were interviewed according to the measurement content of the scale. Referring to the opinions of the executives and combining them with the practice, the relevant measurement items were optimized and adjusted. Finally, 50 executives were selected for pre-research, and the questionnaire items were revised through the reliability and validity test. Considering the privacy and face perception of the enterprise survey, the measurement of each variable in the questionnaire is conducted by anonymous self-assessment measurement, and the questionnaire is suitable for use in an informal environment. All items were assessed using Likert 5-point scales, ranging from 1 (strongly disagree) to 5 (strongly agree).

### Executive cognitive ability

In this study, the scale developed and tested by Morgeson et al. (2005) was used to examine the discernment and decisiveness of executives. It contains four items such as “executives have a strong ability to identify useful information” and “executives are good at seizing opportunities.” In this study, Cronbach's alpha for the four-item scale was 0.887.

### Business model innovation

This paper draws on the scale of Amit and Zott (2001), which classifies business model innovation into efficiency and novelty. Efficient innovation is a valuable acquisition logic based on cost leadership strategy, focusing on the efficiency of innovation performance. There are seven measurement items: “business model reduces production costs, the business model makes it easier for enterprises and external partners.” Novelty innovation is a kind of value acquisition logic based on a differentiation strategy, focusing on the realization of new value in the innovation process. The scale has eight measurement items: “enterprises introduce new partners in the business model, enterprises continuously change and innovate the business model.” In this study, Cronbach's alpha for the scale was 0.902.

### Environmental dynamism

We use Kohli and Jaworski's (1990) scale to measure environmental dynamism, including five items, such as “technology changes rapidly in the industry” and “new technology applications make new product ideas in the industry become a reality.” In this study, Cronbach's alpha for the five-item scale was 0.887.

### Entrepreneurial bricolage

This study draws on the scale that Senyard et al. (2009), which contains eight measurement items. For example, “Enterprises can find new solutions when facing new challenges, and companies can integrate existing resources to face challenges” and so on. In this study, Cronbach's alpha for the eight-item scale was 0.887.

### Control variables

This paper refers to the research of Tihanyi et al. (2000) and Orlando et al. (2002), selects executive age, executive education background, age of the enterprise (year), and enterprises size as control variables. Enterprise size measured by the number of employees (“Under the number of 20” =1; “21–30” =2; “31–40” =3; “41–50” =4; “more than51” =5); “education” (“Under the high school” =1; “high school” =2; “Bachelor's degree” =3; “Graduate degree” =4; “Doctorate degree” =5); “year” (“Under the year of 2” =1; “2–4” =2; “4–6” =3; “6–8” =4; “8 years of age or older” =5); “age” (“Under the age of 25” =1; “26–30” =2; “31–35” =3; “36–40” =4; “41 years of age or older” =5).

### Data analysis

The data analysis of this study is mainly completed through the following three processes. Firstly, the software Amos 24.0 was used to test the discriminant validity among ECA, ED, EB, and BMI. Secondly, SPSS24.0 was used to analyze the correlation between the variables. Finally, structural equation modeling and hierarchical multiple regression analysis were used to verify the proposed hypotheses (Baron and Henry, 2010).

## Results

### Confirmatory factor analysis

Because the data of ECA, ED, EB, and BMI all come from the same subject, there may be a problem of homologous variance. This study mainly uses two methods to solve this problem: First, harman single factor analysis was carried out. After exploratory factor analysis of the measurement items of the four variables (Podsakoff et al., 2003), in the unrotated factor analysis matrix, the total variance factor explanation rate of the first principal component is 38.05%, and there is no serious problem of data homology variance. The total variance explained by all factors whose eigenvalues are greater than 1 is 59.6% (Murtagh and André, 1988). Secondly, Amos 24.0 was used to conduct confirmatory factor analysis (see Table 1). According to the factor analysis results, each variable's measurement items were simplified, and the factor loads of the retained items were greater than 0.5 (Hair et al., 2014). The data reported by Table 1 shows that the four-factor model is superior to the remaining models (χ^2^/df = 1.921; GFI = 0.921; AGFI = 0.906; CFI = 0.967; RMSEA = 0.025; TLI = 0.958).

**Table 1:** The result of confirmatory factor analyses.

**Model**	**Factor**	**χ^2^/df**	**GFI**	**AGFI**	**CFI**	**RMSEA**	**TLI**
Four-factor model	ECA + ED + EB + BMI	1.921	0.921	0.906	0.967	0.015	0.958
Three-factor model	ECA + ED + EB,BMI	2.315	0.896	0.873	0.903	0.043	0.938
Two-factor model	ECA + ED,EB,BMI	3.015	0.812	0.789	0.897	0.076	0.899
One-factor model	ECA,ED,EB,BMI	4.568	0.765	1.132	0.789	0.091	0.807

*n=316. χ^2^, chi-square statistic; CFI, comparative fit index; TLI, Tucker–Lewis index; RMSEA, root mean square error of approximation; and GFI, goodness-offit index*.

### Descriptive statistics

Table 2 reports the mean, variance, and correlation coefficients, average variance extraction (AVE), and composite reliability (CR) for all variables included in this study. There was a significant positive correlation between ECA and EB (R = 0.561, *p* < 0.01), BMI (R = 0.531, *p* < 0.01), and ED (R = 0.492, *p* < 0.01). There was a significant positive correlation between EB and BMI (R = 0.431, *p* < 0.01) and ED (R = 0.352, *p* < 0.01). There was a significant positive correlation between ED and BMI (R = 0.391, *p* < 0.01). The above data analysis results provide preliminary proof for the hypothesis verification of this study. All variables had AVE values greater than 0.5 and CR values greater than 0.7. The convergence validity of this variable for each variable is satisfactory (Fornell and Larcker, 1981).

**Table 2:** Correlation and descriptive statistics.

**Var**	**M**	**SD**	**Age**	**Edu**	**Year**	**Size**	**ECA**	**EB**	**BMI**	**ED**
Age	3.181	1.431	1							
Edu	3.822	0.762	−0.251**	1						
Year	1.981	1.241	0.342**	−0.121	1					
Size	4.392	0.492	0.151*	−0.153	0.612**	1				
ECA	3.383	0.493	0.071	−0.087	−0.042	−0.091	1			
EB	3.991	0.582	0.042	−0.094	−0.114	−0.012	0.561**	1		
BMI	3.902	0.561	0.004	−0.036	−0.213**	−0.043	0.531**	0.431**	1	
ED	3.951	0.581	0.023	−0.052	−0.145*	0.442**	0.492**	0.352**	0.391**	1
AVE							0.551	0.561	0.576	0.560
CR							0.872	0.851	0.802	0.801

*p < 0.05 and **p < 0.01.

### Hypothesis testing

#### Mediating effect of entrepreneurial bricolage

We use SPSS 24.0 to examine the mediating role of entrepreneurial bricolage in cognitive ability and business model innovation. The steps are as follows: Firstly, the influence of the control variables selected in the study on the endogenous variables is tested. Secondly, the main effects of the independent and dependent variables are tested. Finally, the mediating effects of the control, independent, and dependent variables are introduced to test the model. The results of regression analysis of executive cognitive ability, entrepreneurial bricolage, and business model innovation are shown in Table 3. Executive cognitive ability has a significant positive impact on business model innovation (β2 = 0.561, *P* < 0.001, M2) (Baron and Henry, 2010). Therefore, hypothesis H1 is supported.

**Table 3:** Hypothesis test results.

	**BMI**				
**Model**	**M1**	**M2**	**M3**	**M4**	**M5**
**Control variables**					
Age	−0.315**	−0.199**	−0.191**	−0.219**	−0.143**(−2.904)
Education	0.051	−0.039	−0.054	0.012	−0.020
Year	0.036	0.023	0.025	−0.043	0.014
Size	0.117*	0.027	−0.035	−0.072	0.034
**Independent variable**					
ECA		0.561***		0.231***	0.18**
**Mediating variable**					
EB			0.546**	0.346**	
**Moderating variable**					
ED				0.17	0.17
**Interaction**					
ED*ECA				0.385***	0.386***
F-value	5.233***	30.060***	28.003***	14.280***	90.341***
R^2^	0.077	0.375	0.359	0.222	0.644
ΔR^2^	0.062	0.299	0.282	0.145	0.567
D-W	1.960	1.960	1.920	1.903	2.045
VIF_max_	1.652	1.697	2.707	1.667	1.704

*p < 0.05, **p < 0.01, and ***p < 0.001.

In testing the mediating effect, we first put the control variables into the regression equation. Secondly, the mediating variable is put into the regression equation. Finally, independent and mediating variables were simultaneously added to the regression equation. From Model 3 in Table 3, we can see that entrepreneurial bricolage has a significant positive impact on business model innovation (β3 = 0.546, *p* < 0.01). It can be seen from Model 4 that after adding both independent variables and mediating variables, the regression coefficient of executive cognitive ability on business model innovation is less but still significant (β4 = 0.231, *p* < 0.01), while entrepreneurial bricolage also has a significant impact on business model innovation (β5 = 0.346, *p* < 0.01). Entrepreneurial bricolage partially mediates the relationship between executive cognitive ability and business model innovation, assuming H2 is supported.

#### Moderating effects of environmental dynamism

The moderating effect is used to analyze the moderating effect of environmental dynamics on the relationship between executives' cognitive ability and business model innovation. We first take the business model innovation as the dependent variable and then add the control variable, independent variable, regulatory variable, and the product of the independent variable and regulatory variable to the regression equation. In order to eliminate the collinearity, we standardized the independent variable and the regulatory variable, respectively, when constructing the product term of the independent variable and the regulatory variable. From Model 5 in Table 3, it can be seen that environmental dynamism plays a significant moderating role between executive cognitive ability and business model innovation (β6 = 0.386, *p* < 0.01). This shows that the more dynamic the environment is, the more significant the relationship between executives' cognitive ability and business model innovation is. Hypothesis H3 is established.

#### Moderated mediation effects

We test the indirect effect of cognitive ability on business model innovation through entrepreneurial bricolage under different degrees of environmental dynamism (plus or minus one standard deviation) and obtain 95% confidence intervals. As the data reported in Table 4 shows, the confidence interval does not contain 0, proving that the conditional process model is valid (Baron and Kenny, 1986). Therefore, hypothesis H4 is supported.

**Table 4:** Test results of conditional process model.

**Moderator**	**LEVEL**	**Effect**	**Standard error**	**95% CI**
ED	HIGH	0.112	0.023	[0.049, 0.161]
	LOW	0.139	0.034	[0.154, 0.273]

*Bootstrap size = 5,000*.

## Discussion

### Theoretical implications

Firstly, our study facilitates the understanding that executives' cognitive ability is a motivating effect on business model innovation. This study explores how the cognitive ability of new venture executives affects business model innovation and provides a reference for the follow-up research. The current research still needs to analyze and discuss the antecedent logic of business model innovation from different perspectives (Zott et al., 2011). This study takes Chinese new ventures as the research object. It explores the role path of executives' cognitive ability on business model innovation from the perspective of cognitive ability, which provides an essential reference for the study of cognitive causes of business model innovation (Osiyevskyy and Jim, 2015). It also further enriches the research on entrepreneurial cognition and business model innovation.

Secondly, our study found that entrepreneurial bricolage is a critical mediating mechanism in executives' cognitive ability-business model innovation. Based on entrepreneurial bricolage theory, executives' cognitive ability could promote entrepreneurial bricolage, which would promote new ventures' business model innovation. Meanwhile, this study empirically tests previous research that executives' cognitive ability is helpful in explaining that executives affect resource bricolage (Baker and Nelson, 2005) and responds to calls for “focus on the universal form of the entrepreneurial bricolage theory” (Senyard et al., 2014).

Thirdly, our study revealed the indirect relationship between executive cognitive ability and business model innovation through entrepreneurial bricolage under the condition of environmental dynamics. This study investigates the boundary effect of environmental dynamism on the relationship between executives' cognitive ability, entrepreneurial bricolage, and business model innovation in new ventures, which provides a reference for explaining managers' perception of the external environment and the impact of environmental dynamism on business model innovation (Osiyevskyy and Jim, 2015). At the same time, it also provides a reference for studying the causes of entrepreneurial action based on practical logic or improvisation (Baron and Ward, 2004). Moreover, this study expands and deepens the research context of entrepreneurial bricolage theory from the perspective of cognitive ability and further enriches the research on entrepreneurial cognition (Sarasvathy, 2004).

### Managerial implications

Our study has got practical suggestions. Firstly, this study proves that new venture executives' cognitive ability positively impacts business model innovation. Managers of new ventures should strengthen the attention and analysis of the business environment, actively improve their cognitive ability through entrepreneurial learning and communication, formulate reasonable planning schemes for business model innovation, and ultimately improve the performance of business model innovation.

Secondly, this study shows that entrepreneurial bricolage is a critical intermediary in executive cognitive ability and business model innovation. New venture managers should know the importance of entrepreneurial bricolage and constantly explore the channels and methods of entrepreneurial bricolage to solve the problem of resource constraints through the creative use of existing resources.

Thirdly, environmental dynamics can promote the relationship between executive cognitive ability, entrepreneurial bricolage, and business model innovation. Executives' cognitive ability is the driving force of business model innovation in new ventures, which is affected by entrepreneurs' factors and the external environment. On the one hand, enterprise managers should strive to enhance the willingness and motivation of enterprise business model innovation. In addition, the government should build a platform linking experts, managers, and universities to facilitate the exchange and cooperation of business model innovation and improve the efficiency of business model innovation of new enterprises through government assistance.

### Limitations and directions for future research

This study has an essential contribution to entrepreneurial bricolage theory and business model innovation antecedents, but there are still some inadequacies. Future research can be deepened and improved in the following aspects: Firstly, the data source of this study is cross-sectional data, which has causal verification defects to a certain extent. Longitudinal or experimental research can be carried out to test each variable's mechanism further. Secondly, this study mainly investigates the senior managers of entrepreneurial enterprises, and other entrepreneurial team members can be included in future research. Thirdly, the research model needs to be further enriched. The business model innovation process is affected by various complex mechanisms. This study only explores the role of cognitive ability, entrepreneurial bricolage, and environmental dynamism. Future research can introduce cognitive mechanisms such as opportunity identification and entrepreneurial learning into the research model to further deepen and expand research.

## Conclusion

Based on the literature review and empirical research tests, we constructed a moderated mediation model and reached the following research conclusions:

First, the direct and indirect effects of the variables were examined. The new venture executives' cognitive ability has a significant positive impact on business model innovation. This shows that the cognitive ability of new venture executives plays an essential role in breaking through the dilemma of business model innovation. Entrepreneurial bricolage is mediating between executive cognitive ability and business model Innovation. This shows that new ventures usually face the dilemma of resource constraints and cannot effectively carry out business model innovation. Entrepreneurial bricolage action is helpful to achieve the combination of resources and promote new ventures to accomplish business model innovation.

Second, the moderating effect is tested. Environmental dynamism positively moderates the relationship between executives' cognitive ability and business model innovation. Environment changes help to strengthen the cognitive ability of enterprises, prompt executives to quickly analyze environmental threats and innovation opportunities, identify technological and market risks, and find the critical focus of innovation, which helps promote new ventures' business model innovation.

Thirdly, the mediating effect with regulation is tested. Environmental dynamism positively moderates the mediating role of entrepreneurial bricolage in cognitive ability and business model innovation. Environmental dynamism fully stimulates the integration and bricolage activities of new venture executives, which helps to overcome the hindrance of resource shortage to business model innovation. This shows that the entrepreneurial bricolage mechanism under environmental dynamics is crucial in the influence path of executive cognitive ability on business model innovation. Only after experiencing the influence of environmental dynamics can business model innovation succeed.

## Data availability statement

The raw data supporting the conclusions of this article will be made available by the authors, without undue reservation.

## Ethics statement

Ethical review and approval were not required for the study on human participants following the local legislation and institutional requirements. The authors declare that they strictly adhered to the APA guidelines on ethical research practices. The patients/participants provided their online informed consent to participate in this study, which stated the voluntary nature of participation and assurance of confidentiality and anonymity.

## Author contributions

DH: conceptualization, methodology, writing-original draft preparation, review, and funding acquisition. AX: theory direction, supervision, and funding acquisition. CL: theory direction, methodology, and draft revisions. All authors have read and agreed to the published version of the manuscript.

## Funding

This study was supported by the National Natural Science Foundation of China, Grant Number 71672101 and Social Science Planning Project of Shandong, Grant Number 21BGLJO2.

## Conflict of interest

The authors declare that the research was conducted in the absence of any commercial or financial relationships that could be construed as a potential conflict of interest.

## Publisher's note

All claims expressed in this article are solely those of the authors and do not necessarily represent those of their affiliated organizations, or those of the publisher, the editors and the reviewers. Any product that may be evaluated in this article, or claim that may be made by its manufacturer, is not guaranteed or endorsed by the publisher.
